# PreLnc: An Accurate Tool for Predicting lncRNAs Based on Multiple Features

**DOI:** 10.3390/genes11090981

**Published:** 2020-08-23

**Authors:** Lei Cao, Yupeng Wang, Changwei Bi, Qiaolin Ye, Tongming Yin, Ning Ye

**Affiliations:** 1 College of Information Science and Technology, Nanjing Forestry University, Nanjing 210037, China; caolei@njfu.edu.cn (L.C.); wangyupeng940714@163.com (Y.W.); yqlcom@njfu.edu.cn (Q.Y.); 2 School of Biological Science and Medical Engineering, Southeast University, Nanjing 210037, China; bichwei@163.com; 3 The Key Lab of Cultivar Innovation and Germplasm Improvement of Salicaceae, College of Forestry, Nanjing Forestry University, Nanjing 210037, China; tmyin@njfu.edu.cn

**Keywords:** lncRNA, tri-nucleotide, feature selection, prediction

## Abstract

Accumulating evidence indicates that long non-coding RNAs (lncRNAs) have certain similarities with messenger RNAs (mRNAs) and are associated with numerous important biological processes, thereby demanding methods to distinguish them. Based on machine learning algorithms, a variety of methods are developed to identify lncRNAs, providing significant basic data support for subsequent studies. However, many tools lack certain scalability, versatility and balance, and some tools rely on genome sequence and annotation. In this paper, we propose a convenient and accurate tool “PreLnc”, which uses high-confidence lncRNA and mRNA transcripts to build prediction models through feature selection and classifiers. The false discovery rate (FDR) adjusted *p*-value and Z-value were used for analyzing the tri-nucleotide composition of transcripts of different species. Conclusions can be drawn from the experiment that there were significant differences in RNA transcripts among plants, which may be related to evolutionary conservation and the fact that plants are under evolutionary pressure for a longer time than animals. Combining with the Pearson correlation coefficient, we use the incremental feature selection (IFS) method and the comparison of multiple classifiers to build the model. Finally, the balanced random forest was used to construct the classifier, and PreLnc obtained 91.09% accuracy for 349,186 transcripts of animals and plants. In addition, by comparing standard performance measurements, PreLnc performed better than other prediction tools.

## 1. Introduction

Long non-coding RNAs (lncRNAs), defined as a transcript with low protein-coding potential over 200 nucleotides in length, are initially considered as a “noise” of transcription because the expression level and sequence conservation of them are lower than those of messenger RNAs (mRNAs) [1]. However, in recent years, accumulating evidence indicates that lncRNAs exist widely in eukaryotes and are essential elements of the transcriptome [2]. For instance, some lncRNAs have the cis or trans function of possessing gene expression [3], and they can regulate gene expression at multiple levels by complementing homologous sequences of RNA or DNA, or by forming molecular frameworks and scaffolds assembled through structural macromolecular complexes [4,5,6]. In the gene expression network, some lncRNAs act as important regulators, regulating the nuclear structure and transcription of the cell nucleus, mRNA stability, translation and cytoplasmic post-translational modifications [7]. It is precisely because of the various specific expressions of lncRNAs in organisms that the annotation of the sequences has fallen behind. Therefore, it is of great biological significance to identify lncRNAs from multitudinous sequences.

The transcriptome analysis shows that more than 90% of the eukaryotic genome is transcribed, but only 1% to 2% of the genome can encode proteins, which indicates that it is quite crucial to evaluate the protein-encoding ability of transcripts [8,9]. Similar to the structure of mRNAs, lncRNAs have a poly tail and promoter structure after splicing [10]. Since lncRNAs and mRNAs have certain similarities and both have biological functions [11], the identification and differentiation of lncRNAs and mRNAs deserve further study. Moreover, previous studies have shown that some lncRNAs expressed with high abundance are poorly conserved, while some low-expressed lncRNAs have important functions [12]. Therefore, it is less feasible to seek the regularity directly from the stability of lncRNAs, the protection of lncRNAs between species, and the expression level of lncRNAs [12,13].

With the continuous development of sequencing technology and a large number of species being sequenced, researchers can predict lncRNAs from abundant candidate transcripts with scientific calculations. Since the recognition of lncRNAs and RNA from transcripts is a binary classification problem, many machine learning algorithms have been applied to diverse methods, such as CPAT [14], PLEK [15], CPC2 [16], lncRScan-SVM [17], LncFinder [18], PLncPRO [19], RNAplonc [20], etc. CPAT is a tool for evaluating protein-coding ability based on the linear regression model, which is as popular and species-neutral as CPC2. CPC2 uses a support vector machine (SVM) model with the standard radial basis function kernel, which can rapidly predict the protein-coding ability of sequences and provide an online website. PLEK adopts an improved k-mer scheme and support vector machine algorithm to separate lncRNAs from mRNAs. Compared to the input sequence-based method described above, LncRScan-SVM relies on GTF files of query transcripts and focuses on predicting lncRNAs. LncFinder identifies long non-coding RNA utilizing sequence intrinsic composition, structural information, and physicochemical property, which is released as an R package and a web server. PLncPRO and RNAplonc are tools for predicting plant lncRNAs, providing identification models of monocotyledonous and dicotyledonous plants. In combination with the RNA-seq experiment, thousands of transcripts can be generated, but it is difficult and time consuming for researchers to filter thousands of lncRNAs predictions. Predictive software allows researchers to select the most likely candidate sequences for experimental validation, which will help researchers perform functional verification of lncRNAs more efficiently. However, the general applicability and convenience of the method still need to be improved.

Many methods rely on various genetic identification databases and other sequence alignment files, leading to a complex prediction process that takes up a lot of time and space. In this paper, we propose a convenient and accurate tool “PreLnc”, which uses high-confidence lncRNA and mRNA transcripts to build prediction models through feature selection and classifiers. The false discovery rate (FDR) adjusted *p*-value and Z-value were used for analyzing the tri-nucleotide composition of transcripts of different species. Combining with the ranking features through the Pearson correlation coefficient, we use the incremental feature selection (IFS) method and the comparison of multiple classifiers to build the model. Finally, the balanced random forest was used to construct the classifier, and the model training PreLnc can effectively deal with the imbalance of lncRNAs and mRNAs. Besides, by comparing standard performance measurements with other prediction tools, PreLnc performed better in many aspects and obtained 91.09% accuracy for 349,186 transcripts of animals and plants. Its open-source package is available at https://www.github.com/LeiCao97/PreLnc.

## 2. Materials and Methods

### 2.1. Datasets

The datasets in this paper are mainly from six species, which can be divided into two major categories, one consisting of animals (humans, mice, and cows), and the other consisting of 3 common plants (*Arabidopsis thaliana, Oryza sativa*, and *Zea mays*). For animal datasets, we rigorously filtered long non-coding and protein-coding transcripts with ‘transcript_biotype’ as ‘lncRNA’ and ‘protein_coding’, respectively, from the Ensembl (v97) database (http://asia.ensembl.org/) [21]. Due to the absence of known lncRNAs in some existing plants, long non-coding transcripts of plants were obtained from the GreeNC database, which is a repository of lncRNAs annotated in plants and algae specifically [22]. We selected protein-coding transcripts as negative samples from the EnsemblPlants (v44) database [23]. In order to effectively establish the model, we used CD-hit [24] and set parameters (c = 0.9, aS = 0.9) to filter out highly similar 19,181 lncRNAs and 75,673 mRNAs. For model training and testing, we further divided the datasets into training sets and testing sets, which were randomly sampled from complete datasets (see Table 1). All the datasets were independent of each other and are available online at https://github.com/LeiCao97/PreLncData.

### 2.2. Feature Selection and Extraction

Given that the features play a crucial role in the prediction, we selected a variety of features based on the definition, structure and composition of lncRNAs and mRNAs, which can be directly obtained by scientific calculations. Since each selected feature affects the prediction performance, we selected 11 important features as the first candidate subset through analyzing existing lncRNAs prediction methods, which can be divided into four categories (see Table 2). First, sequence length, GC content and standard deviation (SD) of stop codon counts (SCC) (see Equations (1) and (2)) are the basic features directly derived from transcripts [16].

(1) $$\bar{x}=\frac{1}{3}\overset{3}{\underset{i=1}{\sum }}SCC_{i}$$

(2) $$SD=\sqrt{\frac{1}{3}\overset{3}{\underset{i=1}{\sum }}{\left(SCC_{i}-\bar{x}\right)}^{2}}$$

where $\bar{x}$ represents stop codon counts of three frames ($SCC_{1},\;SCC_{2},\;SCC_{3}$). Second, the structural features are composed of open reading frame (ORF) integrity and some parameters related to the coding sequence (CDS) (CDS length, CDS score and CDS percentage) [17,25], which can be evaluated by txCdsPredict program from UCSC [26]. Theoretical isoelectric point (PI) and length of a predicted peptide, which are related to peptides and calculated by the “ProtParam” module in BioPython [27], were selected as features of the third category. The fourth category is composed of two simple and functional definition features that distinguish protein-coding and non-coding transcripts. Fickett TESTCODE score was used to evaluate combination effects based on nucleotide composition and codon usage bias (see Equations (3)–(8)) [28], whereas hexamer score was selected to evaluate the dependencies between adjacent amino acids (see Equation (9)) [14].

(3) $$N_{\mathrm{content}}=\mathrm{Proportion}\;\mathrm{of}\;\mathrm{nucleotide}\;N\epsilon \left\{A,\;T,\;C,\;G\right\}$$

(4) $$N_{1}=\mathrm{Number}\;\mathrm{of}\;A^{’}s\;\mathrm{in}\;\mathrm{positions}\;1,4,7,\cdots$$

(5) $$N_{2}=\mathrm{Number}\;\mathrm{of}\;A^{’}s\;\mathrm{in}\;\mathrm{positions}\;2,5,8,\cdots$$

(6) $$N_{3}=\mathrm{Number}\;\mathrm{of}\;A^{’}s\;\mathrm{in}\;\mathrm{positions}\;3,6,9,\cdots$$

(7) $$N_{\mathrm{pos}}=\frac{\mathrm{MAX}\left(N_{i}\right)}{\mathrm{MIN}\left(N_{i}\right)+1}\;i\epsilon \left\{1,2,3\right\}$$

(8) $${\mathrm{Fickett}}_{\mathrm{score}}=\overset{8}{\underset{i=1}{\sum }}p_{i}w_{i}$$

in Equation (3), $N_{\mathrm{content}}$ means the proportion of nucleotide Nϵ{A, T, C, G}. In Equations (4) to (7), $N_{1},N_{2},N_{3}$ measure the asymmetry in the distribution of each base among the three codon positions and $N_{\mathrm{pos}}$ measures the deviation of each base from one codon to another. Hexamer score can be calculated by the following equation.

(9) $${\mathrm{Hexamer}}_{\mathrm{score}}=\frac{1}{m}\overset{m}{\underset{i=1}{\sum }}\log\left(\frac{F\left(H_{i}\right)}{F^{\prime }\left(H_{i}\right)}\right)$$

where $F\left(h_{i}\right)$ and $F^{\prime }\left(h_{i}\right)\left(i=0,1,\ldots ,4095\right)$ represent in-frame hexamer frequency, which can be measured by a log-likelihood ratio between coding and non-coding training data sets, respectively.

In RNA molecules, each of the adjacent tri-nucleotides is defined as a codon, representing an amino acid during protein synthesis [29]. Considering that classification performance remains a major concern, we proposed to add a subset of features for animals and plants, respectively, by taking tri-nucleotides as another candidate combinations, which performed well in distinguishing lncRNAs and mRNAs [30,31]. It is worth mentioning that the transcripts between plants and animals or even between each species have certain differences. Therefore, it is meaningful to analyze and select uniformly valid features among 64 tri-nucleotides to enhance classification performance. According to the proportion of each trinucleotide in the transcript, we conducted the significance test of the statistical hypothesis test [32]. *p*-value is a method for statistical test data to fall within the range of error probability, and it is set as a threshold value to measure the significance (≤5% is significant, ≤1% is quite significant). The false discovery rate is often applied to multiple test corrections to the *p*-value, while the FDR adjusted *p*-value is more rigorous to measure its significance [33]. The corresponding Z-value can intuitively reflect the degree of difference, which can be a good measure of the correlation between the inner K-mer of the sequence and the category of structure or even function [34]. The adjusted *p*-value and Z-value were applied to analyze the rules of tri-nucleotides in lncRNAs and mRNAs of different species (see Equations (10)–(13)) [35], to analyze the consistent tri-nucleotides between animals, as well as plants.

(10) $$Z=\frac{X_{1}-X_{2}}{\sqrt{\frac{S_{1}^{2}}{n_{1}}+\frac{S_{2}^{2}}{n_{2}}}}$$

(11) $$S=\sqrt{\frac{\sum {\left(X_{i}-\bar{X}\right)}^{2}}{n-1}}$$

(12) $$P_{\mathrm{value}}=2{P(Z}_{C}\geq \left|Z\right||\;\mu =\mu_{0})$$

(13) $$\mathrm{Adjust}-P_{\mathrm{value}}\left(i\right)=P_{\mathrm{value}}\left(i\right)\times length\left(P\right)/rank\left(P\right)$$

where $X_{1},X_{2}$ represent the averages of a certain tri-nucleotide in the positive and negative sample set, $S_{1},S_{2}$ represent the standard deviation, and $n_{1},n_{2}$ represent the numbers of lncRNAs and mRNAs. In equation 12, *p*-value is the probability that the test statistic ($Z_{C}$) is greater than or equal to the test statistic value calculated based on the observed sample set (|Z|) when the test data is lncRNA ($\mu =\mu_{0}$). In equation 13, *p*-values are sorted from small to large, and the adjusted *p*-value is obtained by calculating the current *p*-value ($P_{\mathrm{value}}\left(i\right)$) multiplied by the total number length(P) divided by the sorting number rank(P). In order to select the tri-nucleotides with the strongest influence as the features, we set the FDR adjusted *p*-value at 1% and ranked the significance of the absolute value of Z-value.

Feature selection method usually has a great influence on the whole research process, thus pearson correlation coefficients (PCCs) [36] and incremental feature selection methods [37] were used to study the correlation between features and transcript types. Firstly, we use the Pearson correlation coefficient to rank the features and remove redundant features (|r|>0.8) to ensure their independence from each other. Then, combining all the min-max scaling normalized feature parameters from training sets, an incremental feature selection method was adopted to conduct efficient classification based on the ranking features and classifiers, which was evaluated with the 10-fold cross-validation [37,38]. Moreover, we analyzed the recognition bias of these features, such as amino acids corresponding to tri-nucleotides that may have an important effect on protein synthesis, for mRNAs of different species. Feature extraction was mainly conducted through modified Python scripts from three known software (CPAT [14] used for Feature 3, CPC2 [16] used for Feature 4–7 and lncRScan-SVM [17] used for Feature 8–11).

### 2.3. Model Foundation and Evaluation

**Figure 1:**
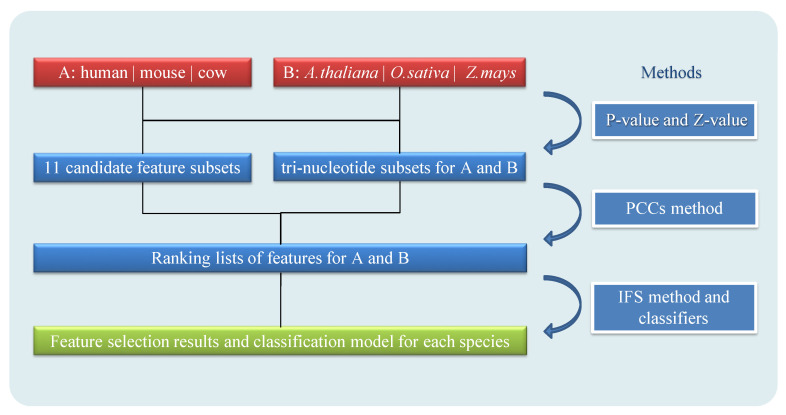
Model architecture of PreLnc. A presents animals (humans, mice, and cows) and B presents plants (*A. thaliana*, *O. sativa* and *Z. mays*). The FDR (false discovery rate) adjusted P-value and Z-value are used to select tri-nucleotides as candidate feature sets. Pearson correlation coefficients (PCCs) are used to obtain ranking lists of features for feature selection results with the incremental feature selection (IFS) method. Model for each species is based on feature selection results and machine learning classifiers.

Feature selection and different classifiers will affect the final prediction performance. Therefore, we combined the incremental feature selection method with a variety of machine learning classifiers, including logistic regression (LR) [39], SVM [40], decision tree (DT) [41], random forests (RF) [42], and K-nearest neighbor methods (KNN) [43]. Based on python’s ‘sklearn’ package, parameters of the 10-fold cross-validation, including sensitivity (SEN) and F-measure (F) (see Equations (14) and (15), were evaluated to build a model with good prediction performance and high applicability. Taking into account the imbalance of the training sets, we divided the positive and negative samples into the data set into different proportions, and finally determined the classifier to balance the random forest. In the overall modeling process, all features were extracted from the transcripts of each species for individual training, and lncRNAs prediction model was created (see Figure 1). All input files are in FASTA format and do not rely on other gene annotation files.

The performance of PreLnc was compared to several mainstream and scalable tools, including CPC2, CPAT, PLEK, LncFinder, PLncPRO and RNAplonc to evaluate the rationality and validity of the model. Standard performance measurement methods were used to evaluate the parameters (see Equations (14)–(18)), including SEN, specificity (SPE), accuracy (ACC), Matthews correlation coefficient (MCC), and area under curve (AUC) characteristics of receiver operating characteristic (ROC) curve through python script. Where TP, TN, FP, FN represent true positive, true negative, false positive, false negative.

(14) $$\mathrm{SEN}=\frac{\mathrm{TP}}{\mathrm{TP}+\mathrm{FN}}$$

(15) $$F=\frac{2\mathrm{TP}}{2\mathrm{TP}+\mathrm{FP}+\mathrm{FN}}$$

(16) $$\mathrm{SPE}=\frac{\mathrm{TN}}{\mathrm{TN}+\mathrm{FP}}$$

(17) $$\mathrm{ACC}=\frac{\mathrm{TP}+\mathrm{TN}}{\mathrm{TP}+\mathrm{TN}+\mathrm{FP}+\mathrm{FN}}$$

(18) $$\mathrm{MCC}=\frac{\mathrm{TP}\times \mathrm{TN}-\mathrm{FP}\times \mathrm{FN}}{\sqrt{\left(\mathrm{TP}+\mathrm{FP}\right)\times \left(\mathrm{TP}+\mathrm{FN}\right)\times \left(\mathrm{TN}+\mathrm{FP}\right)\times \left(\mathrm{TN}+\mathrm{FN}\right)}}$$

## 3. Results

### 3.1. Analysis of Feature Selection and Model Foundation

#### 3.1.1. Analysis of Tri-Nucleotide Differences Among Species

**Figure 2:**
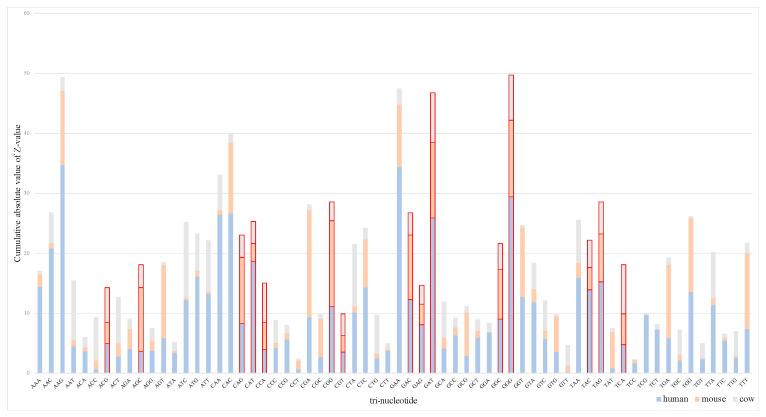
Z-value stacking histograms on animals. The red-labeled bars indicate the ability to distinguish trinucleotides with significant effects from lncRNAs (Long non-coding RNAs) on humans, mice, and cows (adjusted *p*-value < 0.01).

**Figure 3:**
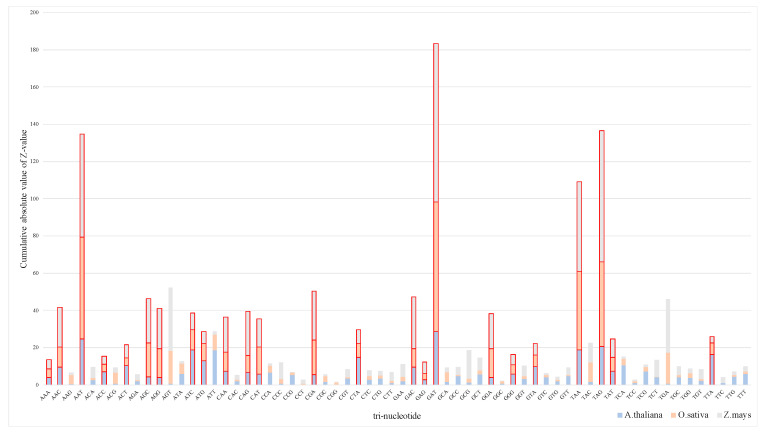
Z-value stacking histograms on plants. The red-labeled bars indicate the ability to distinguish trinucleotides with significant effects from lncRNAs on *A. thaliana, O. sativa* and *Z. mays* (adjusted *p*-value < 0.01).

We used Z-value stacking histograms to show the tri-nucleotide differences among species and the effect of tri-nucleotides on protein-coding ability (see Figure 2 and Figure 3). The Z-value fragment of the same trinucleotide shows the significant degree of differentiating transcripts between species. The difference in the Z-value stacking value of different trinucleotides shows the difference in the transcript sequence itself, which implies that the composition of lncRNA in plants is significantly different. In Figure 2, the columnar segmentation shows the range span of Z-value on humans, mice, and cows (0.183~34.656, 0.079~17.809 and 0.08~12.512), indicating that the significance of tri-nucleotides on humans is greater than that on mice and cows. However, Figure 3 shows that the Z-value span is more differentiated among *A. thaliana*, *O. sativa* and *Z. mays* (0.027~28.755, 0.021~69.513 and 0.152~85.182). Accordingly, the significance of the tri-nucleotides on *Z. mays* was greater than that on the other two plants.

A few tri-nucleotides extracted from two datasets were filtered out because adjusted *p*-value does not meet the significance condition, despite the fact that the cumulative absolute value of Z-value of them is relatively high. Consequently, 15 tri-nucleotides were used as another feature subset for animals, and 24 tri-nucleotides were used for plants. As can be seen in the Figure 2 and Figure 3, 15 tri-nucleotides red-labeled acted on animals, namely, ACG, AGC, CAG, CAT, CCA, CGG, CGT, GAC, GAG, GAT, GGC, GGG, TAC, TAG, and TCA, and 24 tri-nucleotides red-labeled acted on plants, namely, AAA, AAC, AAT, ACC, ACT, AGC, AGG, ATC, ATG, CAA, CAG, CAT, CGA, CTA, GAC, GAG, GAT, GGA, GGG, GTA, TAA, TAG, TAT, and TTA, as they can significantly affect the prediction compared with the other tri-nucleotides. Detailed parameters of adjusted *p*-value and Z-value can be downloaded for viewing (Additional File 1: Table S1).

#### 3.1.2. Correlation Analysis and Ranking Lists of Features

**Figure 4:**
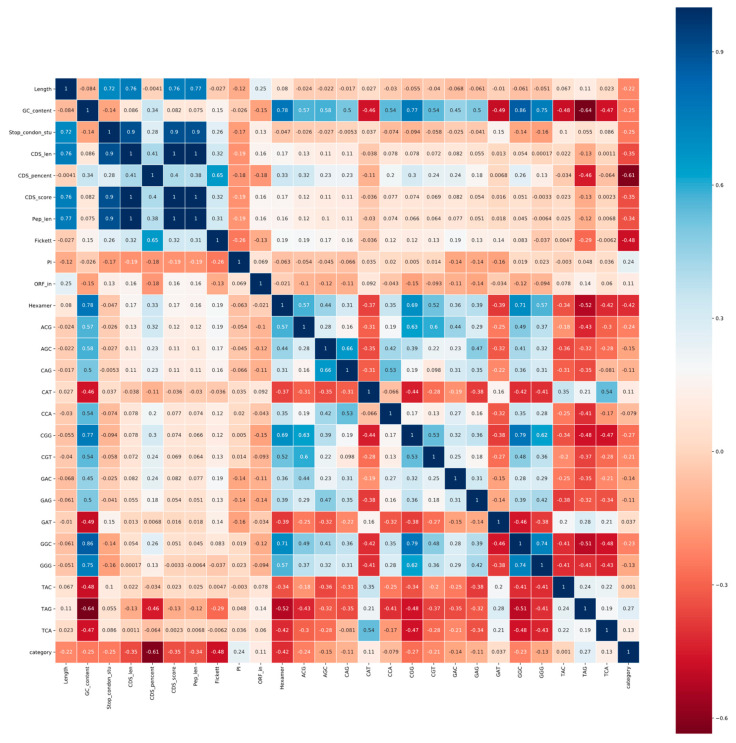
Pearson correlation coefficients of features on animals. The correlation coefficient is in the form of a heat map between various features and transcript categories. Blue represents a positive correlation and red represents a negative correlation. The darker the color, the stronger the correlation. The description of the feature abbreviations: Length, sequence length; GC_content, GC content; Stop_condon_std, standard deviation of stop codon counts (TAA, TAG, TGA); CDS_len, CDS length; CDS_percent, CDS percentage; CDS_score, CDS score of txCdsPredict prediction; Pep_len, peptide length; Fickett, fickett score; PI, isoelectric point; ORF_in, open reading frame integrity; Hexamer, hexamer score, and the other features—tri-nucleotides. The numerical value of the grid indicates the correlation between these two features. The dark blue area in the upper left corner of the figure shows the high correlation over 0.9 between stop codon counts, CDS length, CDS score and peptide length.

**Figure 5:**
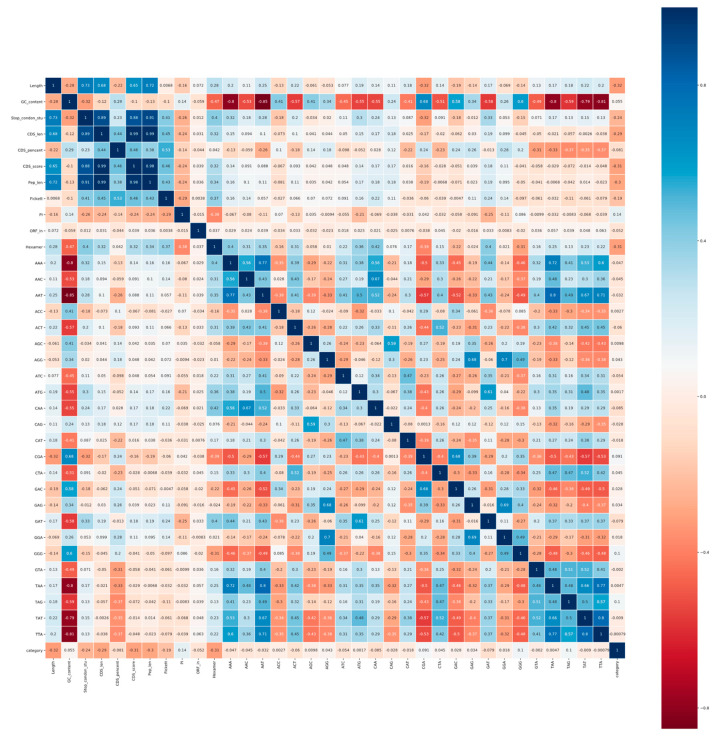
Pearson correlation coefficients of features on plants. The correlation coefficient is in the form of a heat map between various features and transcript categories. Blue represents a positive correlation and red represents a negative correlation. The darker the color, the stronger the correlation. The description of the feature abbreviations: Length, sequence length; GC_content, GC content; Stop_condon_std, standard deviation of stop codon counts (TAA, TAG, TGA); CDS_len, CDS length; CDS_percent, CDS percentage; CDS_score, CDS score of txCdsPredict prediction; Pep_len, peptide length; Fickett, fickett score; PI, isoelectric point; ORF_in, open reading frame integrity; Hexamer, hexamer score; and the other features, tri-nucleotides. The numerical value of the grid indicates the correlation between these two features. The dark blue area in the upper left corner of the figure shows the high correlation over 0.88 between stop codon counts, CDS length, CDS score and peptide length.

The Pearson correlation coefficient can not only measure the correlation between individual characteristics and transcription classes, but also eliminate redundant characteristics. Therefore, we enumerated the correlation coefficients of all features in the form of heat maps (see Figure 4 and Figure 5).

As can be seen from Figure 4 and Figure 5, for both animals and plants, four features, namely, standard deviation of stop codon counts, CDS length, CDS score, and peptide length, are highly positively correlated with each other (|r| > 0.8). According to the definition and calculation of highly correlated features, we filter out CDS length and peptide length to ensure the independence between features. The standard deviation of the stop codon count that measures the deviation of the three frames in the ORF is retained, since not all ORFs can be expressed as protein products.

**Figure 6:**
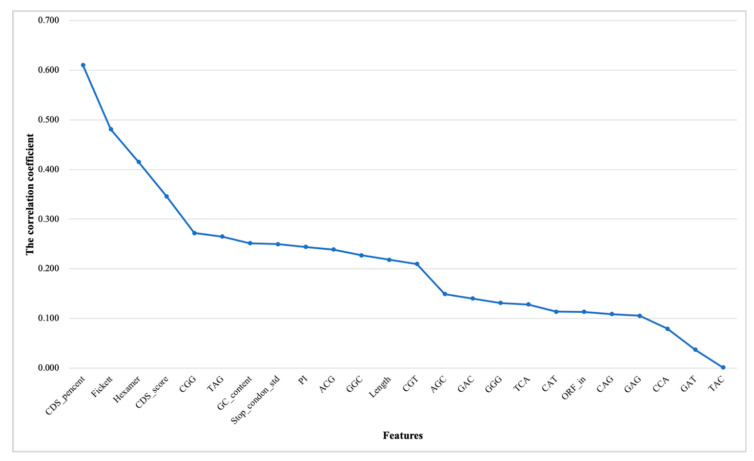
Ranking correlation coefficients (|r|) of features for animals. The horizontal coordinates represent the features. The vertical coordinates represent the correlation coefficient between the features and the transcription category. The description of the feature abbreviations: CDS_percent, CDS percentage; Fickett, fickett score; Hexamer, hexamer score; CDS_score, CDS score of txCdsPredict prediction; GC_content, GC content; Stop_condon_std, standard deviation of stop codon counts (TAA, TAG, TGA); PI, isoelectric point; Length, sequence length; ORF_in, open reading frame integrity and the other features—tri-nucleotides.

**Figure 7:**
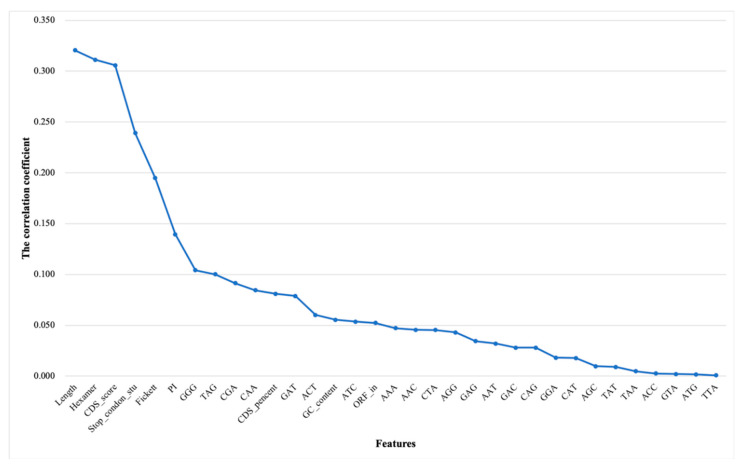
Ranking correlation coefficients (|r|) of features for plants. The horizontal coordinates represent the features. The vertical coordinates represent the correlation coefficient between the features and the transcription category. The description of the feature abbreviations: Length, sequence length; Hexamer, hexamer score; CDS_score, CDS score of txCdsPredict prediction; Stop_condon_std, standard deviation of stop codon counts (TAA, TAG, TGA); Fickett, fickett score; PI, isoelectric point; CDS_percent, CDS percentage; GC_content, GC content; ORF_in, open reading frame integrity and the other features—tri-nucleotides.

According to the importance of individual features in distinguishing lncRNAs from mRNAs, we ranked features to prepare for feature selection (see Figure 6 and Figure 7). On the whole, the features of animals are easier to distinguish from transcripts than plants. Although the sorted list cannot intuitively indicate the impact of features on predictive classification, it is certain that the predictive model of animals will be more versatile than that of plants.

#### 3.1.3. Results of Incremental Feature Selection Method with Multiple Classifiers

**Figure 8:**
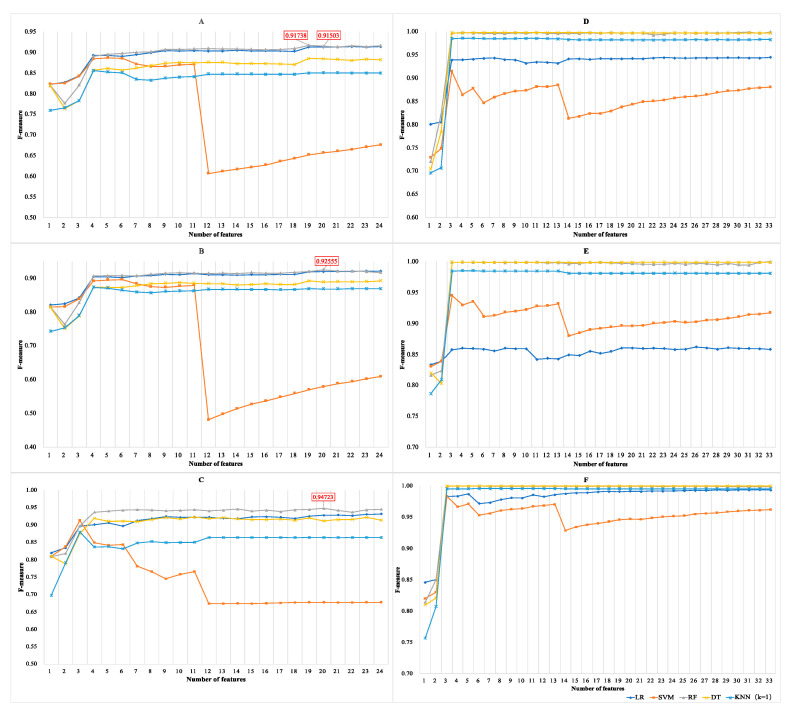
Results of IFS with multiple classifiers. The horizontal and vertical coordinates represent a number of features and F-measure, respectively. (**A**) F-measures on humans. The best parameter is 0.91783 with 19 features and random forest classifier; (**B**) F-measures on mice. The best parameter is 0.92555 with 20 features and random forest classifier; (**C**) F-measures on cows. The best parameter is 0.94723 with 20 features and random forest classifier; (**D**) F-measures on *A. thaliana*. F-measures were basically stable at 0.99 with ranking features; (**E**) F-measures on *O. sativa*. F-measures were basically stable at 0.99 with ranking features; (**F**) F-measures on *Z. mays*. F-measures were basically stable at 0.99 with ranking features.

To ensure that the model can better predict lncRNAs and mRNAs, we integrated the results of the feature selection and classifiers on animals and plants to unify the final standards of the model. Figure 8 shows the dynamic change of F parameters in combination with features and multiple classifiers. In general, a random forest was selected as the final classifier because its performance on six species is significantly better than other classifiers. As far as animals are concerned, the F parameter (0.91503) of the top 20 features of humans are slightly lower than that (0.91738) of the top 19 features and the consistency between species. The top 20 features with high F parameters (0.91503 for humans, 0.92555 for mice and 0.94723 for cows, respectively) were finally used to build animal prediction model (see Figure 8A–C). Compared with animals, random forests have a good classification effect on plants, and the F parameter is generally higher than 0.99 (see Figure 8D–F). The results of the random forest algorithm with ranking features demonstrated good stability on plants, thus we retain all features to further strengthen the model’s balance and generalization ability. The detailed data of SEN parameters and F parameters can be downloaded for viewing (Additional Files 2 and 3: Tables S2 and S3).

**Figure 9:**
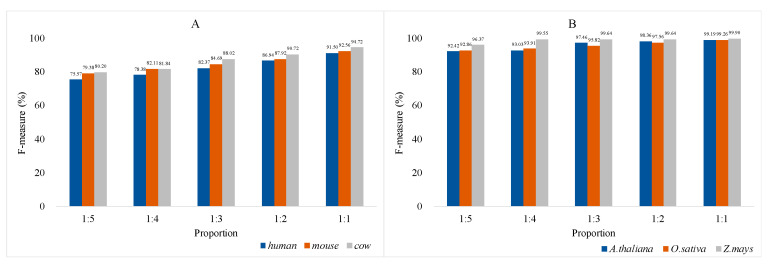
F-measures of balanced random forests in positive and negative sample sets of 1:5, 1:4, 1:3, 1:2, and 1:1, respectively. The horizontal and vertical coordinates represent proportion and F-measure, respectively. (**A**) Balanced random forests with 20 features on animals. Balanced random forests perform poorly on uneven animal datasets with F-measure gaps of 15.93% for humans, 13.18% for mice, and 14.52% for cows; (**B**) Balanced random forests with 33 features on plants. F-measure gaps of plants are smaller (6.77% for *A. thaliana*, 6.40% for *O. sativa*, and 3.53% for *Z. mays,* respectively.

In most plant lncRNA databases, lncRNAs transcript data of many species have yet to be mined, resulting in an imbalance in the number of lncRNAs and mRNAs data. Considering that users can build prediction models for any species using the PreLnc tool, balanced random forests were performed for automatic feature selection [44,45]. We divided the positive and negative samples of the training set into different proportions for comparison (see Figure 9). Balanced random forests perform poorly on uneven animal datasets with F-measure gaps of 15.93% for humans, 13.18% for mice, and 14.52% for cows. Different from animals, F-measure gaps of plants are smaller (6.77% for *A. thaliana,* 6.40% for *O. sativa*, and 3.53% for *Z. mays*, respectively). Therefore, for the common absence of lncRNAs on plants, our method shows considerable advantages.

### 3.2. Comparison and Analysis of Prediction Results

In this paper, the performance of PreLnc is evaluated by comparing standard measurement parameters with some common prediction tools, including CPAT, PLEK, CPC2, and LncFinder, as well as some plant tools, including PLncPRO and RNAplonc. We mainly analyzed the balance and bias of the tool’s prediction performance with SEN, SPE, ACC, MCC and AUC scores. The AUC scores of LncFinder were not listed because it directly determines the type of transcripts. MCC scores were lower, due to the large data difference between the positive and negative sample sets, but it did not affect the comparison between the various tools.

**Figure 10:**
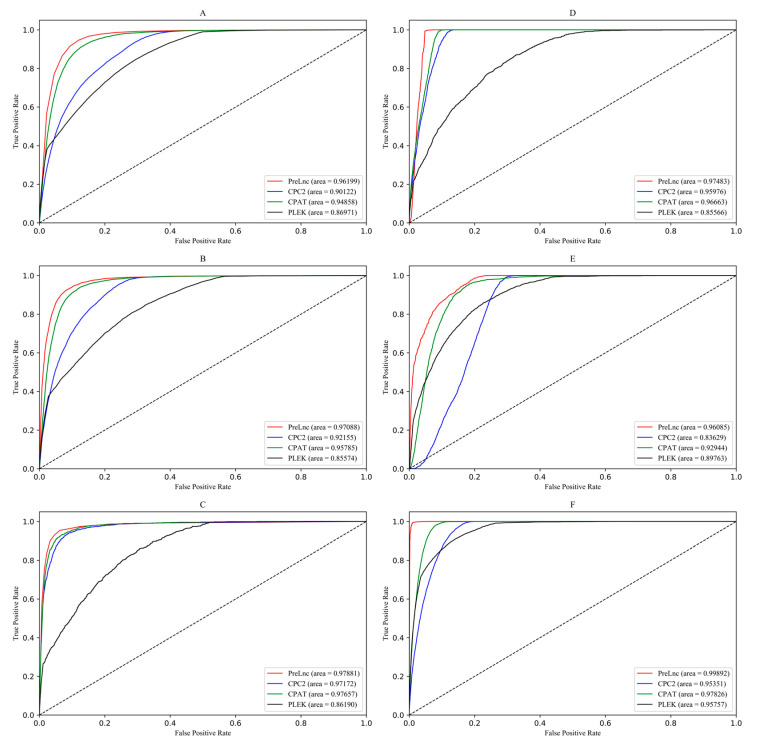
Receiver operating characteristic curves of 6 species. Different colored curves correspond to different tools (red: PreLnc, blue: CPC2, green: CPAT, black: PLEK). The horizontal and vertical coordinates represent the true positive rate and the false positive rate, respectively. (**A**) Receiver operating characteristic on humans; (**B**) Receiver operating characteristic on mice; (**C**) Receiver operating characteristic on cows; (**D**) Receiver operating characteristic on *A. thaliana*; (**E**) Receiver operating characteristic on *O. sativa*; (**F**) Receiver operating characteristic on *Z. mays*.

As a result, PreLnc had a significant advantage in many aspects, especially for SEN, ACC, and MCC (see Table 3). PreLnc has the highest prediction performances, especially for humans, mice, *A. thaliana* and *Z. mays*, but it lacks in prediction performances for cows and *O. sativa*. Additionally, ROC curves were used to represent the AUC scores and authenticity of these methods, showing the great performance of PreLnc (see Figure 10). PreLnc obtained 91.09% accuracy for 349,186 transcripts of animals and plants. On the whole, PreLnc has a higher recognition rate and maintains a balance in distinguishing lncRNAs and mRNAs.

### 3.3. Prediction on Other Known Datasets

Non-coding regulatory RNA is one of the hotspots in life science research, and a large number of lncRNA data and resource databases have been accumulated to facilitate our query and research. To verify the effectiveness of our tools, we further compared the predicted results with other known datasets. We first compared the humans (lncRNAs: 6142, mRNAs: 7485), mice (lncRNAs: 10638, mRNAs: 6460), and *A. thaliana* (lncRNAs: 2562, mRNAs: 13986) datasets from CPC2, which includes mRNA from the RefSeq database with protein sequences annotated by Swiss-Prot and non-coding transcripts from the Ensembl (v87) and EnsemblPlants (v32) databases. PreLnc performed well on the humans and *A. thaliana* datasets, but its predictive effect on mice was slightly lacking (see Table 4). CPAT obtained higher SEN scores on mice and *A. thaliana*, while LncFinder had an advantage in predicting accuracy on mice.

The second dataset was non-coding transcripts of humans and mice from NONCODEv5 [46]. According to the prediction results, PreLnc had higher prediction accuracy for 172,216 humans ncRNA transcripts, reaching 95.319% (see Table 5). CPAT was relatively more accurate (97.594%) in predicting ncRNA transcripts on mice. In general, the prediction results are highly consistent with the above experiments.

### 3.4. Analysis of Model Universality and Generalization Ability

**Figure 11:**
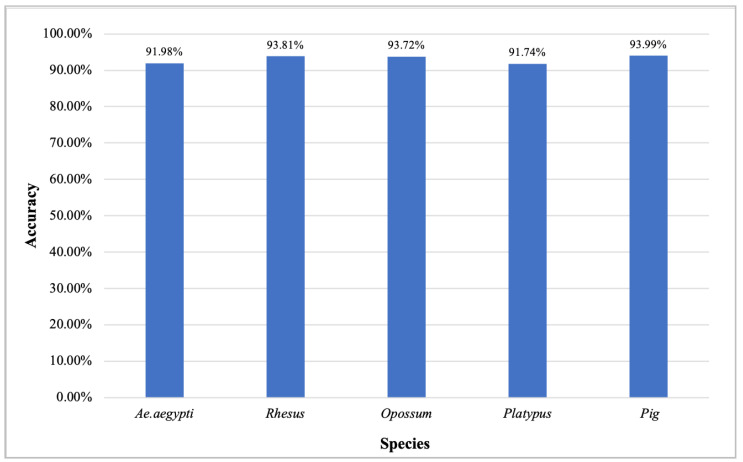
Prediction performance on *Aedes aegypti*, Rhesus, Opossum, Platypus and Pig. The horizontal and vertical coordinates represent species and accuracy, respectively. The 4274 lncRNAs of *A. aegypti* were derived from the systematic research project on the Nucleotide Sequence Database (NT) of NCBI. The lncRNAs of Rhesus (9128 lncRNAs), Opossum (27167 lncRNAs), Platypus (11210 lncRNAs) and Pig (29585 lncRNAs) were derived from NONCODE v5.

In the above comparative experiments, our algorithm predicted a total of 91.09% prediction accuracy on model organisms. In order to further verify the universality and generalization ability of the model, we evaluated and analyzed the prediction effects of other species’ lncRNAs, including *Aedes aegypti*, Rhesus, Opossum, Platypus, and Pig. The 4274 lncRNA transcripts of *A. aegypti* were derived from the systematic research project on the Nucleotide Sequence Database (NT) of NCBI, while other species data were derived from NONCODE v5 [46]. *A. aegypti* lncRNA research covered 117 RNA-seq libraries, and sequencing reads with the average quality score (Phred Score) above 20 were retained for downstream analysis [47]. Compared with model organisms, trained humans models are applied to these less-studied organisms. In Figure 11, the results show that the prediction accuracy of Rhesus, Opossum and Pig are all greater than 93%, and that of *A. aegypti* and Platypus are all greater than 91%. The prediction results of other species verify the effectiveness of PreLnc for predicting novel lncRNAs and its generalization ability.

### 3.5. Comparison of Time Consumption

We compared the system computing time consumption of several tools on the same platform. The configuration is Linux, Ubuntu 16.04.6 LTS 64 bit, Intel® X®(R) CPU E5-2682 v4 @ 2.50 GHz and 2 GB memory. The computational time of the three CPC2 data sets was listed. As seen from Table 6, CPC2, CPAT, RNAplonc and PLncPRO all support fast computing performance. In contrast, PreLnc takes longer than LncFinder, nearly 0.5 times longer, while PLEK takes slightly longer than PreLnc. Meanwhile, the time difference between humans and mice indicates that PLEK may have a certain preference for sequence length.

## 4. Discussion

There are still many problems to be solved in the process of dealing with prediction. For the diversity of species, higher compatibility methods need to be developed under the premise of ensuring the accuracy of prediction. The specificity and relevance of multiple species can be explored in conjunction with effective scientific calculations to extract some of the more biologically significant features.

In this paper, we use a cross-species method to identify lncRNAs and study the intrinsic tri-nucleotide differentiation between animal and plant transcripts. Based on the adjusted *p*-value and Z-value analysis (see Figure 2 and Figure 3), we found that the Z-value span of tri-nucleotides varies more between plants than between animals, which is consistent with evolutionary conservation and the fact that plants are under evolutionary pressure for a longer time than animals [48,49]. Taking codons into account, every three adjacent nucleotides in an RNA molecule are grouped together to represent a certain type of amino acid during protein synthesis [29]. Therefore, in terms of mRNA transcripts, it is meaningful to analyze the corresponding amino acids from the significant differentiation of tri-nucleotides [29,30]. During protein synthesis, the amino acids of the above six species are prominent in threonine, serine, glutamine, histidine, arginine, aspartic acid, glutamic acid, glycine, tyrosine and stop codons (UAG). Besides, there is still a tri-nucleotide corresponding amino acid named proline that is more important for mRNA transcripts of animals. Different from animals, lysine, asparagine, isoleucine, leucine, valine, methionine (starting codon AUG), termination code (UAA) play a major role in the protein synthesis of plants.

As for iterative incremental feature selection, the classification effect of individual features between species is presented in the process. The CDS percentage (Feature: CDS_pencent) prediction effect significantly exceeds 0.8 except for the KNN classifier on animals (see Figure 8A–C). In terms of plants, the transcript length (Feature: Length) has an outstanding effect on the classification of transcripts. The F parameter exceeds 0.70 on *A. thaliana*, and exceeds 0.81 on *O. sativa* and *Z. mays*. Meanwhile, the CDS score (Feature: CDS_score) significantly enhanced the classification ability on all species. Therefore, CDS-related features are more conducive to distinguish between lncRNA and mRNA, which just confirms whether they have protein-coding functions [50].

LncRNA differences between plants and animals were shown as the result of differences caused by imbalance and lack of training data. Judging from the training results of uneven datasets (see Figure 9), prediction models of animals are more dependent on the balance of positive and negative data samples. This may be because the current research on animal lncRNA is more comprehensive, and the mechanism coverage is wider, resulting in the lack of lncRNA datasets and greatly reducing the accuracy of prediction [51,52]. In contrast, the identified plant lncRNA is scarce, and its research field still has a vast, mysterious area [51,52,53]. Therefore, the imbalance of the plant dataset has little effect on the prediction accuracy. 

According to the comparison of the various methods, we found the advantages and biases of these tools in distinguishing RNA transcripts. As seen from the testing results (see Table 3 and Table 4), CPAT and CPC2 can quickly predict the coding ability of transcripts, but the accuracy of CPAT is higher. The poor prediction performance of CPC2 may be due to its prediction only by training human RNA transcripts despite the fact that the transcripts of different species have high complexity and inconsistency. Besides, LncFinder got higher SPE, ACC and MCC scores, especially on cows and *O. sativa*, and the overall classification effect is better than CPAT, CPC2 and PLEK. Especially in terms of plant tools, the poor prediction of PLncPRO may be due to the consensus models for dicots and monocots [19]. At the same time, RNAplonc is biased to differentiate lncRNA especially on *O. sativa*. PreLnc has the best predictive performance on some species, such as humans and *A. thaliana*, rather than cows and *O. sativa*. The lack of species training data and the complexity of the transcript may affect the PreLnc model. In addition, the predicted parameters of animals were lower than those of plants as a whole, which may be the fact that the lncRNAs and mRNAs of animals themselves are more complex and diverse [52,54]. Combined with PreLnc prediction results on other species (see Figure 11), the PreLnc model is proved to have good effectiveness, universality and generalization.

In this study, these results all suggested that lncRNA is less conserved among different species, and transcription sequence was certainly difficult to be used for reference among species [55,56]. Plant lncRNA research is still in its infancy and has great research value, which may reveal unknown new mechanisms that control plant growth and differentiation [53,57]. When studying and analyzing differences in the overall RNA transcripts of animals, a more comprehensive and systematic data structure should be taken to solve the impact of high differentiation. Moreover, further studies can be conducted to compare transcript sequences from functional verification and mechanism studies.

## 5. Conclusions

Nowadays, a variety of tools were developed for lncRNAs prediction, most of which use scientific calculation methods to predict sequences, to accelerate the annotation of unknown genomes for the Human Genome Project. In this work, in addition to screening the tri-nucleotides of species with high genetic similarity as features, other features, such as sequence definition, composition and function, were extracted to propose a more effective method. Compared with other tools, PreLnc can be directly obtained from the transcripts and have certain expansibility, universality and fault tolerance. PreLnc is a convenient and user-friendly tool, but it still needs to be improved in terms of computing speed. On the whole, PreLnc has good predictive performance and supports the prediction of lncRNAs for multiple species.

## Figures and Tables

**Table 1:** Datasets of animals and plants. Transcripts of animals were obtained from Ensembl (v97). Long non-coding and protein-coding transcripts of plants were obtained from GreeNC and EnsemblPlants (v44), respectively. All of the data is filtered by CD-hit, and the datasets are independent of each other.

Dataset	Species		Training	Testing
1	Humans	positive	12,000	24,323
		negative	12,000	80,324
2	Mice	positive	6500	6534
		negative	6500	53,239
3	Cows	positive	1000	976
		negative	1000	36,346
4	*Arabidopsis thaliana*	positive	1400	1339
		negative	1400	33,345
5	*Oryza sativa*	positive	2500	2487
		negative	2500	33,970
6	*Zea mays*	positive	7500	7843
		negative	7500	68,460

**Table 2:** The first subset of 11 features. The features can be classified into four categories: (1) Basic features: No 1, 2, 3. (2) ORF (open reading frame) and CDS (coding sequence) related features: No 4–7. (3) Peptide related features: No 8, 9. (4) Functional definition features: 10, 11.

No.	Feature	Introduction
1	Length	sequence length
2	GC_content	GC content
3	Stop_condon_std	standard deviation of stop codon counts (TAA, TAG, TGA)
4	ORF_in	open reading frame integrity
5	CDS_len	CDS length
6	CDS_score	CDS score of txCdsPredict prediction
7	CDS_percent	CDS percentage
8	Pep_len	peptide length
9	PI	isoelectric point
10	Hexamer	hexamer score
11	Fickett	fickett score

**Table 3:** Methods comparison.

Species	Methods	SEN%	SPE%	ACC%	MCC%	AUC%
*Humans*	*CPAT*	95.321	81.759	84.911	67.763	94.857
	*CPC2*	94.742	69.398	75.288	54.402	90.122
	*PreLnc*	**96.633**	**85.000**	**87.703**	**72.801**	**96.199**
	*PLEK*	87.761	81.219	82.739	61.160	86.971
	*LncFinder*	95.663	84.005	86.714	70.781	/
*Mice*	*CPAT*	**95.883**	86.842	87.831	62.111	96.414
	*CPC2*	94.781	76.235	78.263	47.693	92.155
	*PreLnc*	94.154	**89.927**	**90.389**	66.525	**97.087**
	*PLEK*	91.185	74.534	76.354	43.730	85.574
	*LncFinder*	95.638	89.448	90.219	**66.557**	/
*Cows*	*CPAT*	94.570	91.391	91.474	44.095	97.657
	*CPC2*	87.602	94.759	94.572	50.225	97.172
	*PreLnc*	**95.389**	93.743	93.787	50.768	**97.881**
	*PLEK*	90.164	83.228	83.409	30.043	86.190
	*LncFinder*	93.955	**95.251**	**95.217**	**55.497**	/
*A. thaliana*	*CPAT*	99.851	90.097	90.474	50.910	96.663
	*CPC2*	82.450	92.473	92.086	47.245	95.976
	*PreLnc*	**99.925**	**93.138**	**93.400**	**58.598**	**97.483**
	*PLEK*	88.200	82.876	83.082	34.318	85.566
	*LncFinder*	99.477	90.610	90.953	51.833	/
	*PLncPRO*	73.786	88.925	88.340	35.359	/
	*RNAplonc*	99.776	90.814	91.160	52.444	/
*O. sativa*	*CPAT*	94.692	82.437	83.273	46.333	92.944
	*CPC2*	78.166	77.265	77.327	31.660	83.629
	*PreLnc*	96.220	82.058	83.024	46.697	**96.085**
	*PLEK*	89.867	85.514	85.811	47.849	89.763
	*LncFinder*	94.094	**87.861**	**88.241**	**53.995**	/
	*PLncPRO*	30.197	76.334	73.186	3.849	/
	*RNAplonc*	**99.517**	77.489	78.991	43.352	/
*Z. mays*	*CPAT*	98.126	91.788	92.439	71.936	97.826
	*CPC2*	88.920	89.239	89.206	60.756	95.351
	*PreLnc*	**99.796**	**97.547**	**97.779**	**89.514**	**99.892**
	*PLEK*	90.960	90.879	90.888	65.360	95.757
	*LncFinder*	98.546	92.331	92.970	73.453	/
	*PLncPRO*	66.888	77.615	76.512	30.455	/
	*RNAplonc*	99.308	85.465	86.883	60.881	/

The numbers in bold are the highest parameters of the prediction results. CPAT, CPC2, PreLnc, PLEK and LncFinder predicted lncRNA in all species, and RNAplonc and PLncPRO specifically predicted plant lncRNA. PreLnc has obvious recognition advantages in humans, mice, *A. thaliana* and *Z. mays*, and the AUC value maintains a high score in identifying lncRNAs and mRNAs of 6 species. LncFinder had the best predictive results on cows and *O. sativa*.

**Table 4:** Prediction on CPC2 dataset.

Species	Methods	SEN%	SPE%	ACC%	MCC%
*Humans*	*CPAT*	90.247	95.409	92.574	85.285
	*CPC2*	92.692	95.995	94.181	88.386
	*PreLnc*	**96.072**	**98.893**	**97.344**	**94.705**
	*PLEK*	92.866	89.086	91.163	82.132
	*LncFinder*	93.106	96.239	94.518	89.054
*Mice*	*CPAT*	**97.879**	91.003	93.601	87.154
	*CPC2*	94.892	93.871	94.257	87.972
	*PreLnc*	92.167	92.254	92.221	83.677
	*PLEK*	96.409	85.888	89.986	80.498
	*LncFinder*	96.703	**95.412**	**95.912**	**91.428**
*A. thaliana*	*CPAT*	**99.961**	93.371	94.391	82.777
	*CPC2*	99.649	95.310	95.981	95.981
	*PreLnc*	98.205	**100.000**	**99.722**	**98.936**
	*PLEK*	98.712	86.004	87.972	68.947
	*LncFinder*	96.721	93.443	93.951	80.768
	*RNAplonc*	98.205	94.101	94.737	83.181
	*PLncPRO*	99.141	94.881	95.540	85.552

The numbers in bold are the highest parameters of the prediction results. The datasets consist of humans (lncRNAs: 6142, mRNAs: 7485), mice (lncRNAs: 10638, mRNAs: 6460), and *A. thaliana* (lncRNAs: 2562, mRNAs: 13986). PreLnc had the highest prediction accuracy of 97.344% on humans and 99.722% on *A. thaliana*, and LncFinder had the highest prediction accuracy of 95.912% on mice.

**Table 5:** Prediction on NONCODEv5.

Methods	NONCODEv5_Humans (172,216 Total)	NONCODEv5_Mice (131,697 Total)
PreLnc	**95.319%**	96.315%
CPAT	93.188%	**97.594%**
CPC2	93.946%	95.928%
PLEK	89.321%	95.195%
LncFinder	94.256%	96.913%

The numbers in bold are the highest parameters (accuracy) of the prediction results. Datasets are obtained from NONCODEv5, including humans (172,216 total) and mice (131,697 total). PreLnc had the highest prediction accuracy of humans lncRNA to 95.319%, while CPAT had the highest prediction accuracy of mice lncRNA to 97.594%.

**Table 6:** Comparison of the system computing time consumption.

Methods	Humans (13,627 Total)	Mice (17,098 Total)	*A. thaliana* (16,542 Total)
CPAT	37 s	46 s	39 s
CPC2	**26 s**	**34 s**	**25 s**
PreLnc	269 s	346 s	279 s
PLEK	400 s	307 s	291 s
LncFinder	198 s	212 s	164 s
*RNAplonc*	/	/	34 s
*PLncPRO*	/	/	56 s

The numbers in bold are the fastest time parameters of CPC2. The configuration of the same platform is Linux, Ubuntu 16.04.6 LTS 64 bit, ^®^te^®^) Xeon(R) CPU E5-2682 v4 @ 2.50 GHz and 2 GB memory. Datasets are obtained from CPC2, including humans (13,627 total), mice (17,098 total), and *A. thaliana* (16,542 total). CPAT, CPC2, PreLnc, PLEK and LncFinder predicted lncRNA in all species, and RNAplonc and PLncPRO specifically predicted plant lncRNA. CPC2 supports the fastest speed in the prediction of lncRNA, followed by CPAT, RNAplonc and PLncPRO within 1 min.

## Supplementary Materials

The following are available online at https://www.mdpi.com/2073-4425/11/9/981/s1. Table S1: Detailed parameters of adjusted *p*-value and Z-value; Table S2: F-measure and Sensitivity on humans, mice and cows; Table S3: F-measure and Sensitivity on *A.thaliana*, *O.sativa* and *Z.mays*.
